# *In memoriam* James S. Clegg (1933–2024)

**DOI:** 10.1016/j.cstres.2024.10.003

**Published:** 2024-10-11

**Authors:** Lawrence E. Hightower

**Affiliations:** University of Connecticut, Storrs, CT 06269, USA

James S. (Jim) Clegg passed away at his home in Bodega Bay, California, on September 11, 2024 with his immediate family by his side. He was 91 years old. Jim was the director and professor at UC Davis Bodega Marine Laboratory from 1985 to 1998.

Jim, the son of a British veteran of World War I, was born and raised in Aspinwall, Pennsylvania. He joined the U.S. Army in 1945 and served two years in postarmistice South Korea. He then attended Penn State University on the GI Bill. Jim graduated with honors with a degree in zoology and earned his PhD in biology at Johns Hopkins University in 1961. He obtained a faculty position at the University of Miami in Coral Gables, Florida, where he distinguished himself as an excellent teacher and research scientist. Jim became interested in using *Artemia*, commonly known as brine shrimp, as a model for a group of organisms known as extremophiles. This early interest and work led to his later work on cell stress responses and the role of intracellular water in the environmental adaptation of extremophiles.

In 1985, Jim became the director and professor at UC Davis Bodega Marine Laboratory. During the ensuing years until his retirement in 1998, Jim led fund-raising efforts that expanded the facility into a major marine research station. He tripled the population of the facility and added a library, aquarium, and administrative offices. A new lecture hall was created, and open areas for educational displays were included that allowed the public to understand the results of research activities at the laboratory.

In 2017, I was invited to participate in the dedication of the James S. Clegg Lecture Hall. I was asked to say a few words about Jim’s career. I used the opportunity to tell the assembled audience of longtime friends and colleagues about the remarkable work that Jim was doing as a member of the Cell Stress Society International (CSSI) since his retirement as Laboratory Director. Many in the audience had not heard about his contributions to the addition of environmental research to the topics covered by the CSSI and its journal *Cell Stress and Chaperones*; however, no one who knew Jim was surprised to learn about the energy, enthusiasm, and passion that he brought to the activities of the CSSI.

Jim served 6 years as a senior editor of *Cell Stress and Chaperones*. He came on board just when CSSI was very much in need of an editor with experience in a range of organisms. These organisms are yielding new and valuable information on the interaction of a changing and more stressful natural environment with those organisms, such as extremophiles, that must cope with these changes. In recognition of his excellent efforts as a two-term Senior Editor of *Cell Stress and Chaperones,* Jim was made a senior fellow of the CSSI at our international congress in San Diego in 2019. Jim continued in his role as consulting editor until his passing in 2024.

Jim published over 140 articles about his scientific research. I was very impressed by a review that Jim published in the *Journal of Cell Biology* in 1984 titled “Intracellular Water and the Cytomatrix: Some Methods of Study and Current Views.”^1^ This was the first time that the available information on this topic had been collected in a coherent framework. Jim’s review article directly influenced work in my laboratory years later, determining the effects of D_2_O and glycerol in animal cells responding to a variety of stressors capable of damaging cellular proteins. Jim continued with his interest in the effects on cells of changes in the composition of the cytomatrix with an article published in 2001 in volume 6, issue 2, page 126 of *Cell Stress and Chaperones*: Viner RK and Clegg JS. Influence of trehalose on the molecular chaperone activity of p26, a small heat shock/a-crystallin protein .^2^

**Fig. 1:**
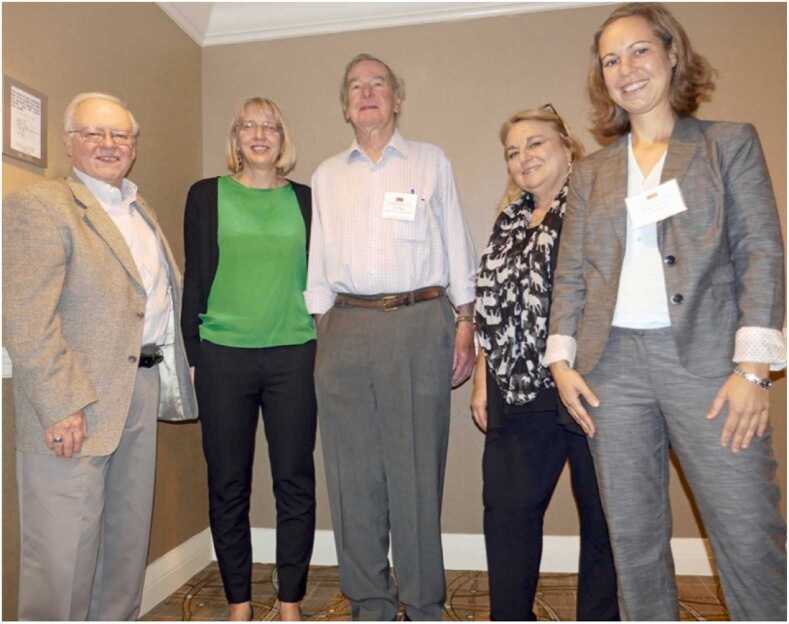
Eighth International Symposium of the CSSI, 2016. Left to Right: Session Co-Chair Larry Hightower, Melody A. Clark (speaker), James Clegg (Senior Editor for Environmental Research, *Cell Stress and Chaperones*) Co-Chair, Rochelle Buffenstein (speaker), and Heike Gruber (speaker).

**Fig. 2:**
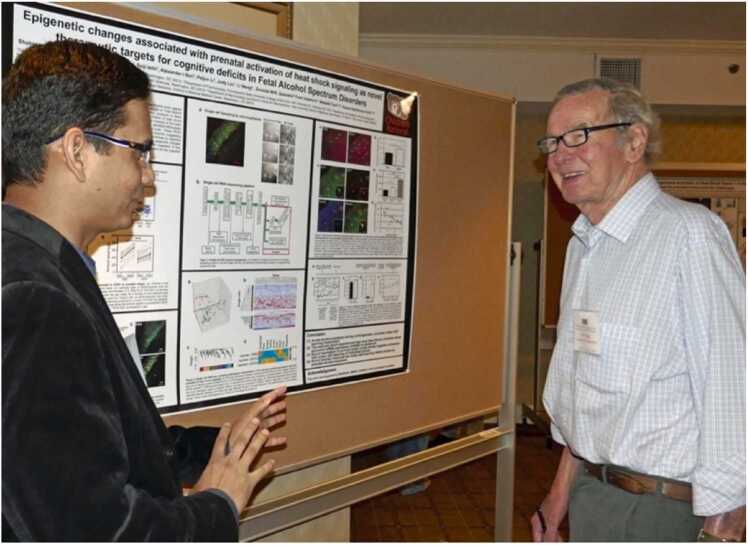
Eighth International Symposium of the CSSI, 2016, poster session. Jim Clegg at the Eighth International Symposium judging a poster presentation by one of the eventual award winners, Mohammad Shahid.

Jim enriched the membership of our society by encouraging students and researchers in various fields of environmental biology to join us. He encouraged them to attend meetings and present their research. In Figure 1, Jim is shown in the center of a group of colleagues who presented at the session he co-chaired in 2016. On his right is Melody Clark, Project Leader, British Antarctic Survey, and winner of the 2022 Polar Medal for her research accomplishments in the Antarctic. Melody joined Jim as senior editors for environmental research on the editorial board of *Cell Stress and Chaperones* when The Environment was added to the subtitle of our journal. Shortly thereafter, Manuela Truebano, lecturer in marine molecular biology and member of the Ecophysiology and Development Research Group at the University of Plymouth, was added as a senior editor as well. I know that Jim was delighted that these accomplished colleagues were added to the editorial board to further enhance the visibility of *Cell Stress and Chaperones* in the study of organisms in a rapidly changing environment. During his retirement, Jim continued to find ways to teach young investigators the fine points of presenting their data. He volunteered to judge poster presentations during our meetings (Figure 2). Graduate students and young investigators continued to benefit from his interest and encouragement.

Jim was a great friend and colleague to many members of the CSSI. At future meetings, we will miss his ever-ready smile, wise counsel, and optimistic view of our future.
